# Gender differences in relation of gender role attitudes and happiness—a mixed-methods research from China

**DOI:** 10.3389/fpsyg.2024.1419942

**Published:** 2024-09-13

**Authors:** Yanan Chen, Xubin Zhang

**Affiliations:** ^1^Faculty of Social Sciences, Lund University, Lund, Sweden; ^2^Department of Politics, School of Philosophy, Zhongnan University of Economics and Law, Wuhan, China

**Keywords:** happiness, gender role attitudes, gender equality, gender differences, China

## Abstract

The gender equality movement represents a monumental advancement in human civilization, liberating countless women worldwide politically, socially, and economically. Intuitively, women are expected to experience greater happiness from the concept of gender equality, while men may see diminished benefits as gender oppression fades away. However, in China, the data indicates a surprising trend: men seem to derive more happiness from gender equality than women. This phenomenon often occurs in countries where gender equality has been achieved, known as the gender equality paradox, while it is relatively rare in countries in transition. In response to this contradiction, a mixed-methods research approach was adopted, utilizing cross-sectional data from the Chinese General Social Survey (CGSS) and conducting interviews with 10 participants. In the context of China, happiness increases with gender-egalitarian attitudes, with men experiencing a more pronounced boost. Upon investigation, Women with egalitarian values, unlike traditional counterparts, are less tolerant of sexual unfairness, limiting women’s happiness growth. Conversely, Chinese men with an equality mindset find greater happiness through benefits like economic relief, reduced family responsibilities, and positive emotional values. To address this incongruity, The government and society should collaborate to overcome the resistance encountered in the practical realization of gender equality, eliminate gender discrimination and opposition, and ensure alignment between the concept and practice of gender equality.

## 1 Introduction

Intuitively, one might expect that women would experience greater happiness from gender equality, as it allows them to break free from feudal patriarchal norms and attain equal economic and social status with men. However, in reality, women generally do not experience substantial increases in happiness from gender equality, while men who hold beliefs in gender equality often report stronger increases in happiness. This counterintuitive phenomenon is recognized in academia as the gender equality paradox. The gender equality paradox has yielded noteworthy findings that defy conventional expectations. Notably, research on this paradox has uncovered an unexpected trend: gender gaps in adolescents’ happiness are more extensive in countries with higher gender equality (Guo et al., 2024). Interestingly, the Guo et al. (2024) study suggests that gender equality disproportionately enhances boys’ happiness more significantly than it does for girls. In this context, the study focuses on China—a developing country with a distinctive blend of modern and traditional norms. The coexistence of these dual influences makes China an intriguing case for studying the relation between gender role attitudes (GRAs), gender equality, and happiness.

Throughout history, Confucian values have profoundly influenced China, prescribing distinct gender roles where men were commonly viewed as breadwinners and family heads, while women were expected to undertake domestic responsibilities (Du, 2001; Zuo, 2002). This entrenched framework permeated various aspects of life, shaping educational pursuits, employment dynamics, and family structures.

After the Chinese Communist Party came to power, the Party promptly initiated a cultural reform based on socialist feminism, to transform societal customs and traditions. In the early 1950s, China widely promoted collectivization, encouraging women to step out of the household and participate in social production from the top down. These measures liberated women’s productive potential but struggled to achieve their emancipation (Yang, 2017). Although collectivism undermined the family as a unit of production, the family’s role in consumption and reproduction remained intact (Du, 2001). Even though gender equality was enshrined in the national constitution, the revolution for women remains at the theoretical level without any working program (Lau, 1996). China’s ideological propaganda on gender division of labor never seemed to challenge the traditional internal and external division of roles (Du, 2001), leaving room for traditional discourse to persist (Yang, 2017). The power dynamics in relations remain unchanged, with men continuing to hold dominance over women (Huang, 1999; Du, 2001). This is evident not only in the gender pay gap in the workplace but also in the perpetuation of traditional female roles within the household (Du, 2001; Jin, 2006). Rather than diminishing, traditional female roles are often reinforced through newspapers, television, movies, and the school system (Lau, 1996), further intensifying the strain of women’s dual responsibilities (Du, 2001; Yang, 2017).

Contemporary China serves as a concentrated microcosm of a transitional country, contending with significant tensions between tradition and modernity regarding gender equality issues. As a Marxist state, China showcases both its most radical and modern aspects, while its millennia-old feudal traditions impart strong conservative tendencies. This interplay of tradition and progressivism is palpable in gender equality matters. Despite the majority of Chinese women (60.5%) having independent careers with well-protected welfare (International Labour Organization, 2024), opportunities for women in career advancement still notably lag behind men. Consequently, men predominantly assume the role of primary breadwinners while women manage household affairs (Zhaopin, 2022; Ellingrud et al., 2023). Moreover, men often face substantial dowry expenses and are tasked with purchasing homes and cars for marriage, reflecting entrenched traditional norms (Yan, 2003). These disparities and conflicts between modernity and tradition contribute to frequent gender-related tensions online (Sun, 2022), posing significant obstacles to advancing gender equality ideals. Therefore, exploring the correlation between gender equality perceptions and happiness in China holds crucial implications for understanding transitional countries more broadly.

In conclusion, GRAs in Chinese society reflect both traditional culture’s influence and modernization’s impact. And because of this, the correlation between gender equality perceptions and happiness in China also exhibits complexity. China is not a highly gender-equal country, yet it is experiencing the gender equality paradox observed in developed countries. The reasons behind this phenomenon warrant further investigation. Prior research using data up to 2015 has suggested the continued existence of the gender equality paradox in China (Zhang and Liu, 2022), yet without delving into its underlying causes or recognizing its prevalence among transitional countries. The current study aims to reevaluate this phenomenon using more recent data and emphasize qualitative methods to understand the complex dynamics and potential contributing factors to this paradox in China.

## 2 Literature review

Research specifically addressing the gender equality paradox in China is currently very limited, which poses challenges for conducting a literature review. However, studies on happiness in China and the increasing research on the relationship between gender equality and happiness are becoming more prevalent. Therefore, in this section, we will focus on reviewing existing studies on happiness in China and the relationship between gender role attitudes and happiness. This will provide a foundational literature review and background knowledge to support the subsequent discussion.

### 2.1 Studies on happiness

In research on happiness in China, the terms happiness, subjective well-being, and life satisfaction are often used interchangeably. Conceptually, happiness and life satisfaction are components of subjective well-being, representing two distinct yet closely related concepts. Happiness emphasizes pleasant emotional experiences, while life satisfaction depends on the respondents’ standards to determine what constitutes a good life (Diener, 1984).

Numerous scales have been designed to measure subjective happiness, including single-item scales (Gurin et al., 1960; Andrews and Withey, 1976) and multi-item scales (Kozma and Stones, 1980; Kammann and Flett, 1983). Single-item scales tend to be less reliable than multi-item scales, but the temporal reliability of single-item measures has been moderately high (Andrews and Withey, 1976; Okun et al., 1984).

Previous studies on happiness in China mainly used public datasets (e.g., Chinese General Social Survey and China Family Panel Studies), which measure happiness using single-item scales. Several sociodemographic characteristics are closely associated with high happiness levels, including being a woman, married, living in urban area, having urban hukou (household registration), and being employed (Knight et al., 2009; Wang and VanderWeele, 2011; Jiang et al., 2012; Liu et al., 2017; Asadullah et al., 2018; Wang et al., 2022; Zhang et al., 2022a; Huang et al., 2023). Age exhibits an inverted U-shaped relationship with happiness (Huang et al., 2023; Shu et al., 2023). Economic factors also influence happiness, with relative income and subjective economic status being stronger predictors than absolute income (Brockmann et al., 2009; Knight et al., 2009; Jiang et al., 2012; Sun and Seo, 2022; Wu and Cao, 2023). Educational attainment may have a positive or negative impact on happiness, with recent studies showing inconsistent results (Asadullah et al., 2018; Jin et al., 2020; Wang et al., 2022; Huang et al., 2023). Additionally, factors such as parenthood, health, social trust, and social capital are closely related to happiness (Knight et al., 2009; Wang and VanderWeele, 2011; Asadullah et al., 2018; Xu and Liu, 2021; Zhang, 2022; Zhang et al., 2022a; Huang et al., 2023).

In general, research on happiness in China has made significant progress and formed a comprehensive knowledge base. However, scholars’ overall attention to the relationship between gender equality perceptions and happiness in China remains limited, in stark contrast to the extensive literature available from Japan, South Korea, and Western countries. Next, let us briefly review the key literature on Gender Role Attitudes and Happiness.

### 2.2 Gender role attitudes and happiness

Gender role attitudes (GRAs) describe views held by individuals regarding the roles men and women should play in society (van der Horst, 2014). It is also known as gender ideology or gender attitudes (Davis and Greenstein, 2009). GRAs broadly encompass many areas, including but not limited to, reproduction, sexual behavior, familial, and work (Bolzendahl and Myers, 2004).

As researchers delve into contemporary society, there is a growing focus on how individuals’ GRAs impact their happiness. Similar to happiness, GRAs are commonly assessed by having respondents report their agreement or disagreement with statements about women’s and men’s responsibilities within the separate spheres (Davis and Greenstein, 2009; Walter, 2018). Davis and Greenstein (2009), in their review of nationally representative surveys, categorized items measuring GRAs into six groups: primacy of the breadwinner role, belief in gendered separate spheres, working women and relationship quality, motherhood and the feminine self, household utility, and acceptance of male privilege. Additionally, Walter (2018) identified nine different aspects, including various combinations of role ascription, conflict, segregation within public and private spheres, and the intersection of these two spheres.

Globally, numerous studies highlight the importance of egalitarian GRAs in shaping individuals’ happiness (Lueptow et al., 1989; Amato and Booth, 1995; Kaufman and Taniguchi, 2006; Kim et al., 2006; van de Vijver, 2007; Kim and Lee, 2008; Taniguchi and Kaufman, 2014; Zhao et al., 2019; Seol et al., 2022). Much of research centers on married couples, with about half of the questions focusing on expectations related to the roles of men and women in married, heterosexual relationships (Davis and Greenstein, 2009).

Initially, studies in Western countries indicated a positive correlation between holding egalitarian beliefs within households and higher happiness, marital satisfaction, and mental health for husbands. Conversely, these beliefs were associated with lower well-being for wives (Lueptow et al., 1989; Amato and Booth, 1995; Kaufman and Taniguchi, 2006). Specifically, women endorsing egalitarian views were more likely to experience decreased marital happiness and stability, leading to higher rates of separation and divorce (Lueptow et al., 1989). However, it’s crucial to acknowledge that some researchers faced challenges in replicating these findings (Crompton and Lyonette, 2005).

Recently, newer studies presented different perspectives, suggesting that adopting more egalitarian GRAs can positively impact the mental health and happiness of both men and women (Kim et al., 2006; van de Vijver, 2007; Kim and Lee, 2008). Additionally, embracing egalitarian GRAs has been shown to mitigate the negative impact of work–family conflict on emotions (Livingston and Judge, 2008).

Although research on the association between GRAs and happiness in the context of China is still in its early stages, scholars have converged on a consistent finding regarding the positive link between egalitarian GRAs and a high level of happiness (Lu and Ruan, 2017; Huang and Guo, 2019; Zhao et al., 2019; Chen et al., 2020; Koo et al., 2020; Wang, 2021; Zhang and Liu, 2022). Egalitarian attitudes toward gender roles contribute to increased self-efficacy in individuals (Chen et al., 2020) and mitigate the risk of depression (Zhang et al., 2022b). Moreover, egalitarian gender norms act as a predictor of family satisfaction and marital stability (Lee, 2022), buffering against the negative impacts of a wife’s income advantage (Zhang, 2015) and work–family conflict (Gong, 2018).

Notably, across various survey datasets, Huang and Guo (2019), Wang (2021), and Zhang and Liu (2022) consistently report that reinforcing egalitarian GRAs leads to increased happiness, with a more pronounced positive impact observed among males. Unfortunately, they stop at presenting the correlation on this issue without delving deeper into the underlying causes.

In general, Western scholars have shown greater interest in the gender equality paradox compared to their Chinese counterparts. Some perceptive Chinese researchers have recognized the existence of a gender equality paradox in China but have not delved deeper into it. Behind this overlooked phenomenon lies an intriguing research question: why does the gender equality paradox, which should theoretically only exist in highly gender-equal countries, manifest in China where gender equality is not fully achieved? Is it because the gender equality paradox is not limited to highly gender-equal countries? Or does the logic behind China’s gender equality paradox differ from the classical understanding of gender equality paradox? If the latter is true, why does China experience a gender equality paradox?

## 3 Materials and methods

The study is divided into two main parts: a quantitative research section and a qualitative research section. The quantitative research section primarily aims to use the latest data to confirm whether the gender equality paradox exists in China. Following the quantitative analysis, we employed qualitative interviews to explore the causes of the gender equality paradox in China, aiming to identify differences between this phenomenon and its Western counterpart. This approach may provide a new explanatory perspective on the gender equality paradox for countries in transition.

This study first used data from CGSS 2021, 2018, and 2017 to verify the existence of the gender equality paradox in China. The specific details of data usage and methods are as follows.

### 3.1 Proposal of hypothesis

To address the uniqueness of the gender equality paradox in China, we first need to use the latest data to confirm that indeed such a paradox exists in China. Based on this, we propose the following two hypotheses to expand the quantitative research section.

*Hypothesis 1*: In China, egalitarian gender role attitudes and happiness significantly positively correlate in men and women.

Firstly, it is crucial to clarify that the gender equality paradox does not imply that holding gender egalitarian views reduces overall happiness. Rather, it suggests that the increase in happiness among women is less significant compared to men. Specifically, a prerequisite for the gender equality paradox is a positive correlation between gender egalitarian views and happiness. In the absence of this correlation, gender equality in a region could be considered very poor, and the gender equality paradox would not be applicable.

Therefore, this hypothesis aims to demonstrate that in China, as a country in transition, there have been substantial improvements in gender equality. Both men and women should experience some degree of increased happiness due to gender equality.

*Hypothesis 2*: In China, the correlation between gender role attitudes and happiness is stronger in men than women.

This hypothesis is intended to verify the existence of the gender equality paradox in China. If hypothesis 1 is fulfilled and hypothesis 2 is also confirmed, it would indicate that China has indeed manifested the gender equality paradox. This substantiates our initial inquiry as a genuine problem of interest, thereby endowing subsequent research with significant value and relevance.

### 3.2 Data source

**Figure 1:**
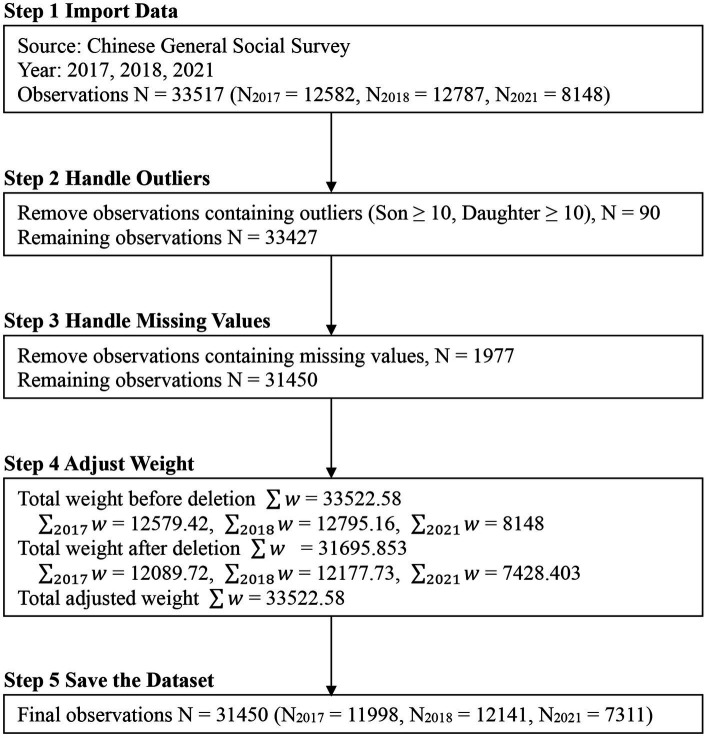
Data cleaning process.

This research utilized the Chinese General Social Survey (CGSS) data from 2017, 2018, and 2021, which is the latest available data for this project. CGSS is China’s earliest national, comprehensive, and continuous large-scale academic social survey project. The survey selected 100 counties (districts) and five major cities—Beijing, Shanghai, Tianjin, Guangzhou, and Shenzhen—as the primary sampling units. It sampled over 10,000 households nationwide, randomly selecting four neighborhood or village committees in each sampling unit, 25 households in each neighborhood or village committee, and then one person from each chosen household for a face-to-face interview. The surveys conducted in 2017, 2018, and 2021 resulted in a total valid sample of 33,517. After pre-processing and removing invalid data, the final sample size for analysis was 31,480. For details on the data cleansing process (see Figure 1).

### 3.3 Measures

#### 3.3.1 Independent variable

The independent variable, Gender Role Attitudes (GRAs), is derived from a five-item gender attitudes scale included in the CGSS questionnaire. Table 1 reports the summary statistics, factor loadings, and Cronbach’s alpha for the original data. In this study, we used four of the original five items to measure GRAs, excluding one item due to its poor performance in Cronbach’s alpha and factor analysis. Each item ranges from 1 to 5, with higher values indicating more egalitarian attitudes and lower values indicating more traditional attitudes.

**Table 1:** Summary, validity and reliability tests for the original data.

Item	Statement	Mean	SD	Factor loading	Alpha
Item 1	Men prioritize careers, while women prioritize family	2.85	1.29	0.65	0.53
Item 2	Men are more competent than women by birth	3.16	1.24	0.68	0.52
Item 3	Marrying a good man is better than doing well at work	3.00	1.24	0.57	0.58
Item 4	During an economic downturn, female employees should be removed first	3.95	0.99	0.51	0.59
Item 5	Couples should share household chores equally	3.89	1.04	0.11	0.72

Obs = 31,402. The results for the 5 items follow the same data cleaning steps as the main study.

Table 2 reports the summary statistics, factor loadings and Cronbach’s alphas for the GRAs scale. We employed principal factor analysis to create a variable summarizing the five items on individuals’ attitudes toward gender roles. The analysis indicated retaining one principal factor (EV = 1.46, Mean = 0, SD = 0.82, Min = −2.02, Max = 1.63) to represent GRAs in this study.

**Table 2:** Summary, validity and reliability tests for gender role attitudes scale.

Item statement	Mean	SD	M-W diff	Min	Max	Factor loading	Alpha
Item 1 – Men prioritize careers, while women prioritize family	2.85	1.29	−0.18	1	5	0.65	0.63
Item 2 – Men are more competent than women by birth	3.16	1.24	−0.09	1	5	0.68	0.61
Item 3 – Marrying a good man is better than doing well at work	3.00	1.24	0.03	1	5	0.57	0.67
Item 4 – During an economic downturn, female employees should be removed first	3.95	0.99	−0.16	1	5	0.50	0.71
Scale (Mean)							0.72
Principal factor score	0	0.82	−0.10	−2.02	1.63		

The factor loadings for all items were considered practically significant, as the suggested minimum value for factor loadings ranges between 0.4 and 0.5 (Guadagnoli and Velicer, 1988; Stevens, 1992; Hair, 1998). Cronbach’s alpha of the scale was 0.72, indicating acceptable internal consistency reliability (DeVellis, 2012).

Overall, respondents tend to oppose gender discrimination in public spheres (Item 4 Mean = 3.95), but maintain traditional attitudes regarding women’s family responsibilities in private spheres (Item 1 Mean = 2.85, Item 2 Mean = 3.16, Item 3 Mean = 3.00). Men exhibited more traditional GRAs compared to women, but the difference between genders averages was minimal (Diff = −0.10).

#### 3.3.2 Dependent variable

The dependent variable, Happiness, is assessed by asking, ‘Overall, do you feel happy with your life?’ with five response levels available (2017, 2018, and 67% of 2021). For the remaining 33% of the 2021 sample, Happiness is measured using the Cantril ladder, which ranges from 0 to 10. The Cantril ladder scores are linearly mapped to a 5-level scale. This adjustment is necessitated by the unique format of the 2021 survey. Single-item measures of happiness have moderate reliabilities, usually between 0.40 and 0.66 (Andrews and Withey, 1976; Krueger and Schkade, 2008). Descriptive statistical results reveal that respondents generally have high-level happiness (Mean = 3.88, SD = 0.83). Over 70 % experienced varying degrees of happiness.

#### 3.3.3 Covariate

Previous research within Chinese samples has indicated that certain socio-demographic and socio-economic variables may serve as predictors of happiness (Brockmann et al., 2009; Knight et al., 2009; Wang and VanderWeele, 2011; Jiang et al., 2012; Liu et al., 2017; Asadullah et al., 2018; Jin et al., 2020; Xu and Liu, 2021; Huang et al., 2023; Shu et al., 2023). Similarly, these variables demonstrate bivariate associations in studies concerning GRAs (Liu and Tong, 2014; Koo et al., 2020; Qian and Li, 2020; Luo, 2021; Wu et al., 2022).

To account for covariates and estimate correlations, we included the following variables as control factors in our models: gender, age, education level, subjective economic status (SES), social trust, self-rated health, marital status, employment status, parenthood status, location, and hukou (household registration identity). Gender is the moderator, with a value of 1 for women and 0 for men. Except for SES, social trust, and health, which were included as ordinal variables, all other variables were incorporated as dummy variables.

Age was recoded into three intervals: 18–39 years, 40–59 years, and 60 years and over. Education was recoded into four categories: Primary or below, Middle school, High school, and College or higher. Responses of “Other” are considered missing values. In the household registration category, responses of “Military,” “No Household Registration,” and “Other” are considered missing values.

Subjective economic status, social trust, and health retained their original ordinal five-category scale, ranging from 1 to 5. Subjective economic status was assessed by asking, “How would you describe your family’s economic status?” with five response options. Social trust was measured by asking respondents, “Do you agree that most people in society can generally be trusted?” Responses were provided on a five-point scale ranging from strongly disagree to strongly agree. Similarly, the health variable was evaluated by asking individuals to rate their current health condition using five degrees.

The definition of unemployed status was based on criteria from the International Labour Organization Bureau of Statistics and International Labour Organization Policy Integration Department (2003). Respondents who were capable of working (never worked after graduation, lost their job due to company or personal reasons, or had their contracted land expropriated), did not work in the past week, actively sought employment in the past 3 months, and were available to start work within 2 weeks were classified as unemployed.

Table 3 presents variable frequency by gender and total. Table 4 summarizes statistics of the original and weighted samples, including means, standard deviations, and gender differences in means.

**Table 3:** Variable frequency by gender and total.

	Man	Woman	Total
Number of observations	14,719	16,731	31,450
Happiness			
Extremely unhappy (=1)	206	231	437
Somewhat unhappy (=2)	875	960	1,835
Neutral (=3)	2,324	2,455	4,779
Somewhat happy (=4)	8,647	9,744	18,391
Extremely happy (=5)	2,667	3,341	6,008
Age			
18–39 years	4,059	4,702	8,761
40–59 years	5,335	6,430	11,765
60 years and over	5,325	5,599	10,924
Education			
Primary or below	4,086	6,520	10,606
Middle school	4,443	4,333	8,776
High school	3,029	2,765	5,794
College or higher	3,161	3,113	6,274
Subjective economic status (SES)			
Low (=1)	1,142	1,226	2,368
Lower-middle (=2)	5,055	5,859	10,914
Middle (=3)	7,345	8,522	15,867
Upper-middle (=4)	1,114	1,084	2,198
High (=5)	63	40	103
Social trust			
Very distrustful (=1)	526	615	1,141
Somewhat distrustful (=2)	2,423	2,801	5,224
Neutral (=3)	1,773	2,312	4,085
Somewhat trustful (=4)	8,283	9,233	17,516
Very trustful (=5)	1,714	1,770	3,484
Health			
Very unhealthy (=1)	551	764	1,315
Somewhat unhealthy (=2)	1,995	2,687	4,682
Normal (=3)	3,556	4,308	7,864
Somewhat healthy (=4)	5,599	6,032	11,631
Very healthy (=5)	3,018	2,940	5,958
Status			
Unmarried	2,380	1,845	4,225
Married	10,888	12,355	23,243
Separated	86	89	175
Divorced	418	388	806
Widowed	947	2,054	3,001
Unemployed	126	71	197
Having children			
No children (=0)	2,500	1,874	4,374
	Man	Woman	Total
Has children (=1)	12,219	14,857	27,076
Hukou/Household registration			
Rural (=0)	8,005	9,365	17,370
Urban (=1)	6,714	7,366	14,080
Location type			
Rural (=0)	5,205	5,675	10,880
Urban (=1)	9,514	11,056	20,570

**Table 4:** Comparison of original and weighted sample statistics.

	Original sample			Weighted sample		
	(Number of obs = 31,450)			(Population size = 33,523)		
	Mean	SD	M-W diff	Mean	SD	M-W diff
Happiness	3.881	0.829	−0.034	3.892	0.808	−0.055
GRAs	0	0.821	−0.097	0.050	0.818	−0.143
Age	51.052	16.987	0.492	44.199	16.365	−0.166
Edu: Primary or below	0.337	0.473	−0.112	0.272	0.445	−0.097
Edu: Middle school	0.279	0.449	0.043	0.320	0.466	0.040
Edu: High school	0.184	0.388	0.041	0.191	0.393	0.046
Edu: College or higher	0.199	0.400	0.029	0.217	0.412	0.010
SES	2.579	0.744	0.013	2.609	0.719	0.008
Social trust	3.540	1.010	0.037	3.486	1.019	0.040
Health	3.516	1.085	0.120	3.697	1.051	0.116
Unmarried	0.134	0.341	0.051	0.178	0.383	0.075
Married	0.739	0.439	0.001	0.749	0.434	−0.048
Separated	0.006	0.074	0.001	0.004	0.066	0
Divorced	0.095	0.294	−0.058	0.048	0.213	−0.033
Widowed	0.026	0.158	0.005	0.021	0.142	0.006
Unemployed	0.006	0.079	0.004	0.009	0.094	0.008
Having children	0.861	0.346	−0.058	0.815	0.388	−0.081
Urban hukou	0.448	0.497	0.016	0.396	0.489	0.017
Urban location	0.654	0.476	−0.014	0.626	0.484	−0.008

Within the original sample, the majority were married (73.9%), urban residents (65.4%), and had children (86.1%). Respondents generally rated their economic status below average (Mean = 2.58), with over 90 % (92.7%) rating their economic status as average or below. Only 20 % (19.9%) had received higher education. Respondents generally exhibited a high level of social trust (Mean = 3.54) and rated their health positively (Mean = 3.52).

After applying the sampling weights provided by CGSS, the average age and the proportion of urban hukou decreased significantly. Additionally, gender differences in happiness and GRAs widened, with women reporting higher levels of happiness and more egalitarian GRAs compared to men.

### 3.4 Methods

#### 3.4.1 Correlation analysis

This study used Stata 17.0 for data cleaning and analysis.

First, non-parametric tests (Spearman’s rank correlation coefficients) were conducted to provide a foundational understanding of the correlations in the data.

Second, ordered logit models were used to analyze both the original and weighted samples. These models considered the interaction term GRAs × Woman and included time fixed effects (M1–M2.2). Further analysis was conducted on the weighted sub-samples by location (rural/urban) and age (3 intervals) (M3.1–M4.3).

Third, accounting for the hierarchical structure of the data, a comparative analysis was performed between ordered logit models with province fixed effects and multilevel mixed-effects ordered logit models with province random effects (M5–M6). The goal of this step was to determine the robustness of the results to different model specifications.

#### 3.4.2 Qualitative analysis

In addition to the quantitative method, the qualitative research method, including interviews, was adopted to explore the unknown fact behind the results of quantitative data analysis. Qualitative data were analyzed through thematic analysis.

The semi-structured interview aims to collect in-depth information about participants’ perspectives, experiences, and beliefs about gender equality. Unlike structured interviews with a fixed set of questions, semi-structured interviews allow for flexibility in conversation, as interviewers can explore further based on interviewees’ responses. Due to the difficulty of reaching geographically dispersed interviewees, we recorded interviews over the telephone to ensure accurate data capture and analysis.

In our research methodology, we utilize semi-structured interviews to explore complex issues related to gender equality and differences. By following the interview outline listed in Table 5, we engage participants in discussions to understand their perspectives on these topics. Through these conversations, we aim to uncover nuanced perspectives and experiences that shape their beliefs about gender equality and differences. By exploring participants’ opinions on which gender benefits more from supporting gender equality, along with their reasons and evidence, we can deepen our understanding of these multifaceted issues.

**Table 5:** Semi-structured interview outline.

Topic	Question
Part A. Gender equality	a. Asking about the definition and understanding of gender equality
	b. Discussing the importance of gender equality
	c. Asking for any experience or observation about gender inequality
Part B. Gender difference	a. Asking about opinions on which gender benefits more from supporting gender equality, in terms of positive impact
	b. Asking about reasons for the response to the previous question
	c. Asking for any experience or observation as the evidence

Ten interviewees were selected (five women and five men) for this qualitative study. They are aged between 25 and 35 years old, with high levels of education (owing Bachelor’s Degree or above). All the participants acknowledge the inherent equality between men and women, even though vary in specific details and schools. They also had personal experiences and perceptions toward gender equality issues, leading to that the interview data can better explain the reasons behind the quantitative results in this study. Table 6 presents the essential information of interviewees. Four types of information are shown: Code name, Gender, Relationship status, and Parenthood. Each type occupies one column from left to right.

**Table 6:** Sample description of interviewees.

Code name	Age	Gender	Relationship status	Parenthood
A	30	Female	Single	None
B	31	Male	Single	None
C	32	Male	Cohabiting	None
D	33	Male	Dating	None
E	34	Female	Married	A son
F	35	Male	Married	A daughter
G	33	Male	Married	A son
H	25	Female	Single	None
I	26	Female	Dating	None
J	25	Female	Single	None

The four types of relationship status: single, dating, cohabiting, and married.

Upon completing the qualitative data collection phase, we engage in a meticulous process of analysis. Utilizing thematic analysis, we systematically code and categorize the interview transcripts (Braun and Clarke, 2006). This method allows us to uncover the underlying meanings, ideas, and concepts regarding gender inequality and gender differences embedded within the narratives shared by the interviewees.

Through our qualitative approach, we aim to capture the perspectives of a specific demographic: young adults aged under 35 who have received higher education. By focusing on this demographic, we seek to gain insights that reflect the experiences and viewpoints of this particular group on gender equality. Ultimately, this targeted approach enriches our research findings by providing a nuanced understanding of how gender equality is perceived and experienced among educated young adults. Additionally, our findings contribute to the broader discourse on gender equality, enhancing societal understanding and facilitating informed discussions on this crucial topic.

## 4 Results

### 4.1 Non-parametric tests

We conducted Spearman correlation analyses among happiness, GRAs, and covariates, and the outcomes are presented in Table 7. The preliminary result suggests a statistically significant positive correlation between egalitarian GRAs and high levels of happiness (*β* = 0.077, *p* < 0.001).

**Table 7:** Spearman’s correlations.

	Happiness	GRAs
GRAs	0.077^***^	
Woman	0.024^***^	0.060^***^
Age (continuous)	0.037^***^	−0.260^***^
18–39 years	0.014^*^	0.264^***^
40–59 years	−0.076^***^	−0.074^***^
60+ years	0.064^***^	−0.173^***^
Edu: Primary or below	−0.075^***^	−0.313^***^
Edu: Middle school	−0.009	−0.041^***^
Edu: High school	0.029^***^	0.124^***^
Edu: College or higher	0.071^***^	0.297^***^
Subjective economic status	0.263^***^	0.134^***^
Social trust	0.207^***^	−0.021^***^
Health	0.205^***^	0.149^***^
Married	0.064^***^	−0.090^***^
Separated	−0.025^***^	−0.007
Divorced	−0.083^***^	0.021^***^
Widowed	−0.010	−0.084^***^
Unemployed	−0.018^**^	0.014^*^
Having children	0.028^***^	−0.204^***^
Urban hukou	0.092^***^	0.230^***^
Urban location	0.055^***^	0.239^***^

^***^p < 0.001, ^**^p < 0.01, ^*^p < 0.05.

Characteristics associated with high levels of happiness include being a woman, having higher educational attainment, being married, having children, possessing an urban household registration (hukou), and residing in urban areas. Additionally, self-reported higher economic status, social trust, and health status are also related to higher levels of happiness. Moreover, age showed a “U”-shaped association with happiness.

Based on the correlations between GRAs and other variables, it can be inferred that young, unmarried women with an urban hukou, living in urban areas, and having received higher education are more likely to report more egalitarian GRAs. This preliminary analysis provides a foundational understanding of the correlations in the data, guiding subsequent model specifications.

### 4.2 Benchmark results

Table 8 reports the ordered logit model regression results for both the original and weighted samples (M1–M2.2).

**Table 8:** Order logistic results for original and weighted samples.

	Original sample		Weighted sample		
	M1	M1.1	M2	M2.1	M2.2
GRAs	0.0987^***^	0.1423^***^	0.1162^***^	0.1729^***^	0.1775^***^
	(0.0161)	(0.0229)	(0.0216)	(0.0300)	(0.0300)
Woman	0.1664^***^	0.1670^***^	0.1841^***^	0.1907^***^	0.1916^***^
	(0.0229)	(0.0229)	(0.0290)	(0.0289)	(0.0289)
Age					
40–59 years	−0.0529	−0.0559	−0.0410	−0.0443	−0.0372
	(0.0327)	(0.0327)	(0.0408)	(0.0407)	(0.0406)
60+ years	0.4965^***^	0.4950^***^	0.5091^***^	0.5071^***^	0.5179^***^
	(0.0389)	(0.0389)	(0.0495)	(0.0495)	(0.0494)
Education level					
Middle school	0.0789^*^	0.0825^**^	0.0849^*^	0.0898^*^	0.1020^*^
	(0.0315)	(0.0315)	(0.0400)	(0.0400)	(0.0399)
High school	0.1066^**^	0.1110^**^	0.1956^***^	0.2012^***^	0.1997^***^
	(0.0381)	(0.0381)	(0.0497)	(0.0496)	(0.0496)
College or higher	0.1431^***^	0.1476^***^	0.2190^***^	0.2258^***^	0.2225^***^
	(0.0429)	(0.0430)	(0.0546)	(0.0547)	(0.0549)
SES	0.5837^***^	0.5833^***^	0.5480^***^	0.5473^***^	0.5481^***^
	(0.0175)	(0.0175)	(0.0219)	(0.0219)	(0.0218)
Health	0.3831^***^	0.3840^***^	0.3859^***^	0.3874^***^	0.3864^***^
	(0.0130)	(0.0130)	(0.0166)	(0.0167)	(0.0166)
Social trust	0.3599^***^	0.3598^***^	0.3288^***^	0.3287^***^	0.3323^***^
	(0.0129)	(0.0129)	(0.0166)	(0.0166)	(0.0166)
Married	0.2432^***^	0.2423^***^	0.1612^*^	0.1624^*^	0.1549^*^
	(0.0563)	(0.0563)	(0.0718)	(0.0716)	(0.0716)
Separated	−0.2102	−0.2111	−0.4063	−0.4017	−0.3848
	(0.1639)	(0.1640)	(0.2230)	(0.2231)	(0.2216)
Divorced	−0.6470^***^	−0.6424^***^	−0.7640^***^	−0.7572^***^	−0.7414^***^
	(0.0839)	(0.0840)	(0.1071)	(0.1072)	(0.1070)
Widowed	0.0858	0.0844	−0.0946	−0.0954	−0.0959
	(0.0709)	(0.0709)	(0.0904)	(0.0902)	(0.0902)
Unemployed	−0.2102	−0.2096	−0.2752	−0.2769	−0.2660
	(0.1395)	(0.1395)	(0.1732)	(0.1728)	(0.1730)
Having children	0.1718^**^	0.1713^**^	0.2141^**^	0.2122^**^	0.2091^**^
	(0.0579)	(0.0579)	(0.0750)	(0.0748)	(0.0748)
Urban hukou	0.1304^***^	0.1301^***^	0.1166^**^	0.1160^***^	0.1151^***^
	(0.0291)	(0.0291)	(0.0361)	(0.0361)	(0.0360)
Urban location	−0.0682^*^	−0.0681^*^	−0.0773^*^	−0.0760^*^	−0.0738^*^
	(0.0294)	(0.0294)	(0.0367)	(0.0367)	(0.0366)
GRAs × Woman		−0.0782^**^		−0.1081^**^	−0.1079^**^
		(0.0297)		(0.0400)	(0.0399)
Time fixed effects	No	No	No	No	Yes

^***^p < 0.001, ^**^p < 0.01, ^*^p < 0.05. Observations = 31,450. Weighted population = 33,523. Values in parentheses are robust standard errors. The control group consists of men under 40 years old who are unmarried, childless, living in rural areas, registered as rural hukou, and have an education level of primary school or below.

Considering socioeconomic and demographic characteristics, our analysis reveals a significant positive correlation between GRAs and happiness (*β* = 0.099, *p* < 0.001). When using the weighted sample and including the interaction effect between GRAs and gender, as well as time fixed effects, the correlation between GRAs and happiness becomes even stronger (*β* = 0.178, *p* < 0.001). This result validates Hypothesis 1, indicating a positive correlation between egalitarian GRAs and happiness for both men and women, consistent with previous studies (Huang and Guo, 2019; Koo et al., 2020; Wang, 2021; Zhang and Liu, 2022; Chen et al., 2023).

Consistent with Hypothesis 2, the significant negative correlation between the interaction term (GRAs × Woman) and happiness highlights gender’s moderating effect on the relationship between GRAs and happiness (*β* = −0.078, *p* < 0.01). In the weighted sample with time fixed effects, the interaction effect becomes stronger (*β* = −0.108, *p* < 0.01). This finding is consistent with previous studies, suggesting that in China, the correlation between GRAs and happiness is stronger in men than in women (Huang and Guo, 2019; Wang, 2021; Zhang and Liu, 2022).

Table 9 reports the regression results for the weighted subsamples categorized by location (rural/urban) and age (three intervals) (M3.1–M4.3). The results for the urban subgroup (M3.2) and the younger subgroup (M4.1) are consistent with the overall sample, showing a significant positive correlation between egalitarian GRAs and happiness (*p* < 0.001) and a significant negative moderating effect of gender on this relationship (*p* < 0.05).

**Table 9:** Order logistic results by location and age group.

	Location type		Age		
	Rural	Urban	18–39 years	40–59 years	60+ years
	M3.1	M3.2	M4.1	M4.2	M4.3
GRAs	0.1013	0.2197^***^	0.3178^***^	0.0778	0.1066^*^
	(0.0520)	(0.0362)	(0.0589)	(0.0427)	(0.0447)
Woman	0.2312^***^	0.1661^***^	0.2277^***^	0.1962^***^	0.0884
	(0.0521)	(0.0362)	(0.0603)	(0.0418)	(0.0461)
Age					
40–59 years	−0.0001	−0.0597			
	(0.0706)	(0.0493)			
60+ years	0.5328^***^	0.5110^***^			
	(0.0843)	(0.0605)			
Education level					
Middle school	0.1594^**^	0.0424	0.0943	0.2129^***^	−0.0542
	(0.0579)	(0.0540)	(0.1134)	(0.0525)	(0.0588)
High school	0.3452^***^	0.1100	0.2539^*^	0.3554^***^	−0.1721^*^
	(0.0948)	(0.0591)	(0.1203)	(0.0686)	(0.0724)
College or higher	0.2719^*^	0.1595^*^	0.3105^**^	0.2948^***^	−0.2417^**^
	(0.1250)	(0.0651)	(0.1182)	(0.0801)	(0.0933)
SES	0.5218^***^	0.5645^***^	0.4213^***^	0.6141^***^	0.6429^***^
	(0.0364)	(0.0272)	(0.0425)	(0.0321)	(0.0338)
Health	0.3581^***^	0.4085^***^	0.4146^***^	0.3979^***^	0.3073^***^
	(0.0252)	(0.0220)	(0.0350)	(0.0245)	(0.0231)
Social trust	0.3242^***^	0.3378^***^	0.3047^***^	0.3405^***^	0.3783^***^
	(0.0285)	(0.0202)	(0.0305)	(0.0236)	(0.0264)
Married	0.1488	0.1622^*^	0.1704	0.1561	0.2165
	(0.1456)	(0.0777)	(0.1031)	(0.1257)	(0.1443)
Separated	0.1123	−0.6961^**^	−0.5318	−0.2995	−0.2424
	(0.4015)	(0.2447)	(0.6114)	(0.2725)	(0.4060)
Divorced	−0.7110^**^	−0.7604^***^	−0.9018^***^	−0.6889^***^	−0.4230
	(0.2146)	(0.1218)	(0.1802)	(0.1655)	(0.2613)
Widowed	−0.0964	−0.0943	−1.1230^*^	−0.6015^***^	0.1571
	(0.1639)	(0.1084)	(0.5367)	(0.1759)	(0.1494)
Unemployed	−0.1460	−0.3156	−0.3113	−0.0302	−1.3283
	(0.3834)	(0.1899)	(0.2206)	(0.2775)	(2.0180)
Having children	0.1216	0.2547^**^	0.2381^*^	0.2418	0.1646
	(0.1553)	(0.0804)	(0.1025)	(0.1439)	(0.2185)
Urban hukou	0.1209	0.1265^**^	0.1012	0.1267^*^	0.1855^**^
	(0.0891)	(0.0405)	(0.0611)	(0.0565)	(0.0655)
Urban location			−0.0905	−0.1120^*^	−0.0145
			(0.0676)	(0.0528)	(0.0610)
GRAs × Woman	−0.0939	−0.1050^*^	−0.1921^*^	−0.0643	−0.0825
	(0.0691)	(0.0500)	(0.0813)	(0.0557)	(0.0610)
Number of observations	10,880	20,570	8,761	11,765	10,924
Weighted population size	12,530	20,992	14,516	12,471	6,535
Time fixed effects	Yes	Yes	Yes	Yes	Yes

^***^p < 0.001, ^**^p < 0.01, ^*^p < 0.05. Weighted sample. Values in parentheses are robust standard errors. The control group consists of men under 40 years old who are unmarried, childless, living in rural areas, registered as rural hukou, and have an education level of primary school or below.

Table 10 presents the ordered logistic regression results with province fixed effects and random effects (M5–M6). Province fixed effects control for unobserved heterogeneity at the provincial level, while province random effects allow for the assessment of between-province variability. The results considering the hierarchical structure of the data (M5–M6) are consistent with the baseline model (M2.2), confirming the reliability and robustness of the model.

**Table 10:** Ordered logistic results with province fixed effects and random effects.

	M5	M6
GRAs	0.1658^***^	0.1664^***^
	(0.0301)	(0.0350)
Woman	0.1778^***^	0.1787^***^
	(0.0291)	(0.0261)
Age		
40–59 years	−0.0445	−0.0452
	(0.0408)	(0.0419)
60+ years	0.4930^***^	0.4924^***^
	(0.0497)	(0.0699)
Education level		
Middle school	0.0496	0.0512
	(0.0403)	(0.0387)
High school	0.1444^**^	0.1458^*^
	(0.0512)	(0.0613)
College or higher	0.1096^*^	0.1130
	(0.0560)	(0.0679)
SES	0.5678^***^	0.5669^***^
	(0.0220)	(0.0366)
Health	0.3925^***^	0.3920^***^
	(0.0169)	(0.0187)
Social trust	0.3228^***^	0.3234^***^
	(0.0167)	(0.0217)
Married	0.1188	0.1203
	(0.0717)	(0.0752)
Separated	−0.4597^*^	−0.4568^*^
	(0.2270)	(0.2119)
Divorced	−0.7831^***^	−0.7820^***^
	(0.1081)	(0.0858)
Widowed	−0.1259	−0.1248
	(0.0912)	(0.1070)
Unemployed	−0.2380	−0.2382
	(0.1692)	(0.1558)
Having children	0.1943^**^	0.1953^*^
	(0.0748)	(0.0843)
Urban hukou	0.0788^*^	0.0808^**^
	(0.0370)	(0.0301)
Urban location	−0.0478	−0.0509
	(0.0383)	(0.0488)
GRAs × Woman	−0.0906^*^	−0.0916^*^
	(0.0401)	(0.0396)
Constant		0.0733
		(0.0182)
Time fixed effects	Yes	Yes
	M5	M6
Province fixed effects	Yes	No
Province random effects	No	Yes

^***^p < 0.001, ^**^p < 0.01, ^*^p < 0.05. Weighted sample. Observations = 31,450. Weighted population = 33,523. Values in parentheses are robust standard errors. The control group consists of men under 40 years old who are unmarried, childless, living in rural areas, registered as rural hukou, and have an education level of primary school or below.

In conclusion, our quantitative analysis of the CGSS data from the past 3 years (2017, 2018, 2021) confirms Hypotheses 1 and 2. Firstly, there is a significant positive correlation between egalitarian GRAs and happiness. However, this correlation is not significant in the rural subgroup and the middle-aged subgroup, which may be due to the stronger adherence to traditional gender role norms in rural areas and the greater economic and life pressures faced by middle-aged individuals. Secondly, the interaction term between GRAs and women shows a significant negative correlation with happiness. This negative correlation is also observed in the urban subgroup and the younger subgroup.

Our results indicate that in China, individuals with egalitarian gender role attitudes are more likely to report higher levels of happiness. Compared to women, men derive greater happiness from endorsing gender equality beliefs. Moving forward, to address theoretical gaps in previous research, we will analyze semi-structured interview data. This qualitative analysis will complement our quantitative results, providing a theoretically grounded explanation based on empirical evidence.

### 4.3 Qualitative results

Since the quantitative study results verified Hypothesis 1 and 2, it is necessary to investigate the reasons for gender differences in the relationship between GRAS and happiness through a qualitative study. Interviews with 10 participants (see Table 6) who held the view of gender equality were then conducted to collect data for further explanation.

#### 4.3.1 Positive incentives of gender equality for happiness

In the interviews, all informants clearly stated they could gain happiness from the concept of gender equality, as indicated in earlier findings (Huang and Guo, 2019; Koo et al., 2020; Zhang and Liu, 2022). When asked about further reasons, the interviewees gave various explanations, including spiritual satisfaction and the complementary relationship.

Firstly, the interviewees all believe that gender equality is a progressive concept, and holding this advanced value of equality brings them great spiritual pleasure.

There is a significant difference in gender equality among the respondents in this study. For example, most interviewees, regarded as libertarian in gender, stress that gender equality requires respect for different gender characteristics (Interviewee E). A few interviewees have the view of strong socialist feminism, believing gender equality is a part of social and economic justice for all people (Interviewee C). One female interviewee holds a radical feminist perspective (Interviewee A). Though the understanding of gender egalitarianism varies among the informants, equality is the common value they espouse:

Gender is innate and inseparable from human characteristics. So, what kind of rights or status people have should not be different due to gender. Everyone should be treated equally in status, rights, interests and opportunities, regardless of gender. (Interviewee E)

Since relevant data to examine the influence of gender equality on happiness is limited, we added a series of questions to challenge their commitment to the idea of equality. For example, “When gender inequality can bring you huge benefits, are you willing to give up gender equality and return to the traditional concept of male–female affiliation?” All the female respondents gave a negative answer that no matter what men can bring them, they are not willing to be subordinate to males. Although men’s answers are not as firm as women’s, most can no longer accept the traditional male-dominance relationship:

Equality itself will bring us a lot, such as equal knowledge and equal experience, which can help families solve many problems, and the feeling of being evenly matched is actually quite good. (Interviewee G)

The concept of equality itself is a great incentive for the participants. Even when faced with a dispute between righteousness and benefit, most interviewees are still willing to stick to gender egalitarianism.

#### 4.3.2 Qualitative analysis of women’s relatively low subjective happiness

The previous data analysis revealed that women who view gender equality as an important value have less happiness than men with egalitarian values.

The main reason for women’s relatively low happiness lies in the disconnection between their cognition of gender equality and the actual practice of sexual egalitarianism in Chinese society. That is to say, the progressive concept of gender equality has not been widely accepted and implemented in China (Wu et al., 2022; United Nations Development Programme, 2023), and women with equal gender values have to face traditional gender discrimination in all walks of life. Traditional women are less likely to perceive gender discrimination because they tolerate or even accept concepts of gender inequality (e.g., “women are inferior to men” and “men dominate outside, women dominate inside”). However, women who believe in gender equality have lowered the tolerance threshold for sexual discrimination. They can capture such kind of discrimination very keenly, feel confused and angry, and then act to resist sexual prejudice:

In high school, there is generally some discussion about what men and women should do or what they are good at. What impressed me most was that when I discussed that with my classmates one afternoon, my dissatisfaction suddenly became an annoyance. I felt like I was humiliated by my gender. Boys can do whatever they want, but girls only can do what boys want them to do… Afterward, your classmates (boys and girls) thought I had a bad temper and were not easy to get along with. (Interviewee A)

Women who hold the concept of gender equality are susceptible to discrimination, partly due to their desire to break the prejudice and pursue equality. For example, some interviewees will try their best to tear off the labels imposed on them by society, such as girls should not speak dirty words, women should be meek and considerate, and women should focus on their families. In many cases, this may come from the persuasion of elders, relatives, friends, or enthusiastic people based on their social experience. From their perspectives, these counsels do not contain any hostility or deliberate discrimination, moreover, they can make life easier for Chinese women. Many Chinese women agree with that, but women with gender equality values feel it is systematic discrimination against women and sometimes invoke feminist theories and national policies to defend themselves. This is undoubtedly a manifestation of social progress, but these kinds of revolt always suffered criticism for not behaving like a woman. The experience of an interviewee can better present this predicament:

My relatives keep telling me that a girl should be a teacher, have a secure job, get married and have kids. But I’ve already decided not to get married or have children. Because of it, my dad and I always start arguing within a few words and have a cold war over my life plans 1–2 times a week. I have tried to have calm conversations, but my dad usually unilaterally exported his point of view to me and ignored mine, so we never had a successful conversation. (Interviewee J)

Other girls also complained about the discrimination encountered in their careers. Males have more opportunities than females in employment, promotion, and other professional achievement. The following case is an example:

Recently, in a chat group of employment I was in, someone was recruiting a clerk, men only. I got angry and said, ‘How dare sexism be so blatant in today’s world’. Then others rounded it up by saying that there are also women-only positions. But the chat group has never released a woman-only position. I’ve seen female-only positions on some job Applications, but it’s pretty obvious in their job announcements that they want the job candidate to show up with obvious sexual innuendo. (Interviewee H)

And:

When you are young, you can’t feel sexual discrimination obviously in working, but once you are over 35 years old, when it comes to promotion opportunities, the negative influence of gender discrimination and inequality may become apparent. (Interviewee I)

The unfair competition in careers leads to women’s systemic vulnerability in income and socio-economic status. To lead a better life, many traditional women tend to place their emphasis on the family and rely on capable husbands for their livelihood (Fang and Walker, 2015). Compared to those women, modern women who are unwilling to be dependent on men need to face greater life pressures, which significantly reduces their subjective sense of happiness.

#### 4.3.3 Qualitative analysis of men’s high subjective happiness

According to a new survey, half of the participants believe that gender equality will harm the interests of men (Olivia and Jessica, 2023). However, most of the interviewees in this research provided different perceptions concerning the influence of gender equality on males’ happiness based on their own experiences. This contributes to analyzing why men have relatively high subjective happiness in the quantitative part. In Chinese society, due to the deep-rooted patriarchy, men have always been in a more advantageous position in competition, enjoying relatively more opportunities and resources. Intuitively speaking, the concept of gender equality may diminish their benefits and subsequently reduce their happiness. However, the quantitative results revealed that holding the gender egalitarian will not harm male happiness but enhance it. Three reasons were discovered in the qualitative study: (1) alleviating economic pressure; (2) reducing family responsibilities; and (3) gaining positive emotional values.

Firstly, because of the traditional economic dependence of women on men, Chinese men usually need to bear more financial responsibilities in marriage, married life, and child-rearing. A typical example of this is the bride price, which includes money, property, or other wealth paid by a groom or his family to the woman or her family during betrothal (Li, 2008). Although the reasons why the participants holding gender equality beliefs are not utilitarian, when they choose partners who share the same gender attitudes, it does indeed reduce their financial burden, significantly enhancing their subjective sense of happiness:

The traditional customs require the male family to bear the bride price or some material things. If men and women are equal, the economic pressure caused by buying houses and providing bride prices will be reduced. (Interviewee C)

And:

This is a very practical problem. If my wife did not work, I definitely can’t accept it, because my family can’t survive financially. (Interviewee F)

Secondly, when both partners are advocates of gender egalitarianism, it significantly reduces the household responsibilities borne by the male partner. In the traditional Chinese Confucian family structure, males not only bear economic responsibilities but are also expected to take responsibility for all decisions, even to support the female partner’s family member. For lower-class males, this is undoubtedly a significant burden:

I don’t want to lower my living standard by marrying my wife and bearing a child. I have my interests to pursue and ideals to chase. In such a family pattern (gender equality), I feel free from heavy family responsibilities and can get positive emotional values. (Interviewee G)

When modern women free men from these timeworn obligations, the family type therefore shifts from male dominance to mutual support, undoubtedly significantly increasing men’s sense of happiness:

If marriage cannot give me positive incentives, I will not get married. My wife and I are independent from each other, and we can encourage and help each other in work or life. (Interviewee G)

Finally, gender equality beliefs can also bring many emotional benefits to men, including a sense of progress due to equality, as well as personal growth within equal relationships. Interviewee C gave us some shreds of evidence:

My girlfriend has told me many experiences and influenced me. Just makes me feel some of my shortcomings. I think gender equality is not just an idea. It is a practice. It is not enough if you believe or support it without practice, by which you will find your actions are different from your expectations. You need to keep improving or changing. (Interviewee C)

## 5 Conclusion

This study focused on a special issue in gender studies: the correlation between gender equality and happiness. Analysis of the CGSS 2017/2018/2021 data affirmed the positive impact of gender equality on happiness, aligning with prior research (Chen et al., 2023). Notably, a significant gender disparity was observed in China, wherein men perceiving gender equality tended to experience greater happiness than women. To present the reasons behind the phenomenon in depth, interviews with 10 participants were conducted, finding that holding the view of gender equality could bring them happiness.

In examining gender disparities in happiness, our findings revealed that, on average, Chinese women reported higher levels of happiness than men, as reported in previous studies (Wang and VanderWeele, 2011; Asadullah et al., 2018; Huang et al., 2023). However, there were nuances in how gender equality influenced their subjective happiness. Specifically, we identified that Chinese women’s perception of gender equality did not always align with the prevailing societal practices, leading to a perceived discrepancy between their beliefs and reality. In contrast, Chinese men derived greater happiness from their endorsement of gender equality, benefiting from its positive outcomes such as alleviating economic pressures, reducing familial obligations, and fostering positive emotional values.

This indicates that while China experiences gender equality similar to highly egalitarian countries, the underlying reasons for this phenomenon differ significantly. In developed nations, the gender equality paradox arises from physiological differences between sexes, diminishing marginal returns, and other factors. In contrast, China’s gender equality paradox stems from the profound tension between ideals and reality. Men who hold gender egalitarian views benefit from socio-economic advantages derived from a society with gender inequality, thereby ensuring high levels of happiness. Meanwhile, women who hold these views confront gender discrimination and face equivalent life pressures as men, significantly undermining the promotion of happiness through gender equality ideals. Furthermore, we believe this issue extends beyond China; all countries undergoing transition from traditional to modern societies inevitably encounter similar situations. Our research aims to provide insight and inspiration to such regions facing comparable challenges.

As we mentioned at the beginning, China, as a typical representative of transitional societies, faces the intertwining of modern institutions and traditional orders. Traditional feudal hierarchies emphasize differences between men and women, constructing gender disparities based on physiological differences and artificially creating gaps in power and status, imposing numerous born responsibilities on women. In contrast, modern society emphasizes principles of equality, highlighting the intrinsic homogeneity of personality among individuals. Gender differences constructed by society fundamentally obstruct the equality of personality. Therefore, promoting gender equality ultimately aims to promote equality among individuals, a fundamental responsibility that all modern nations should undertake.

The prevalence of gender equality has been notably advanced through affirmative action movements in China. Nevertheless, substantial strides are still necessary to realize gender equality in practice. Advocating for gender equality and fostering its widespread acceptance in China is imperative. Such efforts would not diminish the happiness of men and women but instead yield a significant positive impact on overall well-being. Thus, from both the standpoint of pursuing equality and utilitarian perspectives, proactive measures to promote gender equality should be undertaken by the Chinese government and society.

## 6 Research limitations

Firstly, the CGSS data has limitations in availability and completeness. The most recent data is only from the 2017, 2018, and 2021 surveys, and because of COVID-19, the 2021 survey has a large number of missing values compared to the previous years. This may skew the results and limit their generalizability. Future studies should consider alternative data sources or methodologies to address these issues.

Secondly, the measurement of gender role attitudes may not be fully accurate or comprehensive. Gender role attitudes represent a complex and multidimensional concept influenced by cultural, social, and individual factors (Hoxmeier et al., 2024). While this study used CGSS items to measure them, the scale may not capture their full scope. Future research should use more comprehensive tools to better understand these attitudes. Similarly, measuring subjective well-being with a single question does not capture its multidimensional nature, including emotional, psychological, and social components. Using multi-item scales is expected to enhance reliability by aggregating responses across several items (Krueger and Schkade, 2008).

Thirdly, the qualitative research sample lacks representativeness. The study relied on participants who were highly educated and young, which may not reflect the diversity of society. Future research should include a broader range of educational levels, occupational backgrounds, and geographical locations to better capture societal diversity.

## Data Availability

The original contributions presented in the study are included in the article/supplementary material, further inquiries can be directed to the corresponding author.

## References

[ref1] Amato P. R. Booth A. (1995). Changes in gender role attitudes and perceived marital quality. Amer. Sociol. Rev. 60, 58–66. doi: 10.2307/2096345

[ref2] Andrews F. M. Withey S. B. (1976). Social indicators of well-being. New York, NY, US: Springer New York.

[ref3] Asadullah M. N. Xiao S. Yeoh E. (2018). Subjective well-being in China, 2005–2010: the role of relative income, gender, and location. China Econ. Rev. 48, 83–101. doi: 10.1016/j.chieco.2015.12.010

[ref4] Bolzendahl C. I. Myers D. J. (2004). Feminist attitudes and support for gender equality: opinion change in women and men, 1974-1998. Soc. Forces 83, 759–789. doi: 10.1353/sof.2005.0005

[ref5] Braun V. Clarke V. (2006). Using thematic analysis in psychology. Qual. Res. Psychol. 3, 77–101. doi: 10.1191/1478088706qp063oa

[ref6] Brockmann H. Delhey J. Welzel C. Yuan H. (2009). The China puzzle: falling happiness in a rising economy. J. Happiness Stud. 10, 387–405. doi: 10.1007/s10902-008-9095-4

[ref7] Chen H. Peng X. Xu X. Yin Y. (2020). The effect of gender role attitudes on the self-efficacy of the older adults: based on data from the third wave survey of Chinese Women’s social status. Asia Pac. J. Soc. Work 30, 273–287. doi: 10.1080/02185385.2020.1744478

[ref8] Chen L. Wu K. Du H. King R. B. Chen A. Li T.. (2023). Less equal, less satisfied? Gender inequality hampers adults’ subjective well-being via gender-role attitudes. Sex Roles 89, 718–730. doi: 10.1007/s11199-023-01392-8

[ref001] Crompton R. Lyonette C. (2005). The new gender essentialism – domestic and family ‘choices’ and their relation to attitudes. Br. J. Sociol. 56, 601–620. doi: 10.1111/j.1468-4446.2005.00085.x 16309438

[ref9] Davis S. N. Greenstein T. N. (2009). Gender ideology: components, predictors, and consequences. Annu. Rev. Sociol. 35, 87–105. doi: 10.1146/annurev-soc-070308-115920

[ref10] DeVellis R. F. (2012). Scale development: theory and applications. London, England, UK: Sage Publications.

[ref11] Diener E. (1984). Subjective well-being. Psychol. Bull. 95, 542–575. doi: 10.1037/0033-2909.95.3.542, PMID: 6399758

[ref12] Du F. (2001). The historical context of Women’s studies: patriarchy, modernity, and gender relations. Zhejiang Acad. J. 24, 106–111. doi: 10.16235/j.cnki.33-1005/c.2001.01.022

[ref13] Ellingrud K. Shi J. Wang Y. Zhao E. Li Y. (2023). Advancing gender equality in the Chinese workplace. Shanghai, China: Mckinsey. (Accessed April 15, 2024).

[ref14] Fang Y. Walker A. (2015). “Full-time wife” and the change of gender order in the Chinese City. J. Chin. Sociol. 2:4. doi: 10.1186/s40711-015-0006-x

[ref15] Gong Z. (2018). The study on urban residents’ work—family balance and its influencing factors. J. Soc. Work. 31, 87–97.

[ref16] Guadagnoli E. Velicer W. F. (1988). Relation of sample size to the stability of component patterns. Psychol. Bull. 103, 265–275. doi: 10.1037/0033-2909.103.2.265, PMID: 3363047

[ref17] Guo J. Basarkod G. Perales F. Parker P. D. Marsh H. W. Donald J.. (2024). The equality paradox: gender equality intensifies male advantages in adolescent subjective well-being. Personal. Soc. Psychol. Bull. 50, 147–164. doi: 10.1177/01461672221125619 36205464

[ref18] Gurin G. Veroff J. Feld S. (1960). Americans view their mental health: a nationwide interview survey. Oxford, England, UK: Basic Books.

[ref19] Hair J. F. (1998). Multivariate data analysis. Upper Saddle River, NJ, US: Prentice Hall.

[ref20] Hoxmeier J. C. Casey E. A. Carlson J. Willey-Sthapit C. (2024). A critical review of measures of gender equitable attitudes: recommendations for conceptualization and future assessment. Sex Roles 90, 231–249. doi: 10.1007/s11199-024-01441-w

[ref21] Huang Y. (1999). The improvement of Women’s status after the founding of the People’s republic of China. J. Tsinghua Univ. 14, 18–27.

[ref22] Huang S. Guo J. (2019). An empirical study of gender inequality impact on well-being based on “world values survey”. Soc. Sci. Front 42, 35–42.

[ref23] Huang Y. Yi D. Clark W. A. V. (2023). Subjective wellbeing in 21st century China: a multi-level multi-dimensional perspective on urban-rural disparities. Appl. Geogr. 159:103071. doi: 10.1016/j.apgeog.2023.103071

[ref24] International Labour Organization (2024). Labor force participation rate, female (% of female population ages 15+) (modeled ILO estimate). Available at: https://ilostat.ilo.org/data/ (Accessed February 6, 2024).

[ref25] International Labour Organization Bureau of Statistics and International Labour Organization Policy Integration Department (2003). International training compendium on labour statistics: module 1, statistics of employment, unemployment, underemployment: economically active population. Geneva, Switzerland: ILO.

[ref26] Jiang S. Lu M. Sato H. (2012). Identity, inequality, and happiness: evidence from urban China. World Dev. 40, 1190–1200. doi: 10.1016/j.worlddev.2011.11.002

[ref27] Jin Y. (2006). Rethinking the “Iron girls”: gender and labor in China during the cultural revolution. Sociol. Stud. 21, 169–193. doi: 10.19934/j.cnki.shxyj.2006.01.008

[ref28] Jin Y. Li Z. An J. (2020). Impact of education on Chinese urban and rural subjective well-being. Child Youth Serv. Rev. 119:105505. doi: 10.1016/j.childyouth.2020.105505

[ref29] Kammann R. Flett R. (1983). Affectometer 2: a scale to measure current level of general happiness. Aust. J. Psychol. 35, 259–265. doi: 10.1080/00049538308255070

[ref30] Kaufman G. Taniguchi H. (2006). Gender and marital happiness in later life. J. Fam. Issues 27, 735–757. doi: 10.1177/0192513X05285293

[ref31] Kim M. Lee E. (2008). Explorations on the feminism of Korean men on the bases of their social structural variables and its effect on well-being. Korean J. Woman Psychol. 13, 1–18. doi: 10.18205/kpa.2008.13.1.001

[ref32] Kim M. Lee H. Han Y. (2006). Explorations on the feminism of Korean women on the bases of their social structural variables and its effect on well-being. Korean J. Woman Psychol. 11, 83–105.

[ref33] Knight J. Song L. Gunatilaka R. (2009). Subjective well-being and its determinants in rural China. China Econ. Rev. 20, 635–649. doi: 10.1016/j.chieco.2008.09.003

[ref34] Koo A. Hui B. Pun N. (2020). Gender ideologies of youth in post-socialist China: their gender-role attitudes, antecedents, and socio-psychological impacts. Chin. Sociol. Rev. 52, 487–514. doi: 10.1080/21620555.2020.1768366

[ref35] Kozma A. Stones M. J. (1980). The measurement of happiness: development of the Memorial University of Newfoundland scale of happiness (MUNSH). J. Gerontol. 35, 906–912. doi: 10.1093/geronj/35.6.906, PMID: 7440930

[ref36] Krueger A. B. Schkade D. A. (2008). The reliability of subjective well-being measures. J. Public Econ. 92, 1833–1845. doi: 10.1016/j.jpubeco.2007.12.015, PMID: 19649136 PMC2597879

[ref37] Lau S. (1996). Growing up the Chinese way: Chinese child and adolescent development. Hong Kong: Chinese University Press.

[ref38] Lee Y.-J. (2022). Lingering male breadwinner norms as predictors of family satisfaction and marital instability. Soc. Sci. 11:49. doi: 10.3390/socsci11020049

[ref39] Li X. (2008). The bride price in folk customs and its evolution. Folk. Stud. 24, 253–262.

[ref40] Liu A. Tong X. (2014). The present situation of gender attitudes and the factors influencing them: based on the third survey of Women’s social status in China. Soc. Sci. China 35, 116–129.

[ref41] Liu Y. Zhang F. Wu F. Liu Y. Li Z. (2017). The subjective wellbeing of migrants in Guangzhou, China: the impacts of the social and physical environment. Cities 60, 333–342. doi: 10.1016/j.cities.2016.10.008

[ref42] Livingston B. A. Judge T. A. (2008). Emotional responses to work-family conflict: an examination of gender role orientation working men and women. J. Appl. Psychol. 93, 207–216. doi: 10.1037/0021-9010.93.1.207, PMID: 18211146

[ref43] Lu J. Ruan Y. (2017). A study of gender differences in marital matching structure and subjective well-being. J. Fujian Prov. Comm. Party Sch. C.P.C. 29, 74–81. doi: 10.15993/j.cnki.cn35-1198/c.2017.10.013

[ref44] Lueptow L. B. Guss M. B. Hyden C. (1989). Sex role ideology, marital status, and happiness. J. Fam. Issues 10, 383–400. doi: 10.1177/019251389010003005

[ref45] Luo M. (2021). Cohort dynamics in relation to gender attitudes in China. Chin. J. Sociol. 7, 194–216. doi: 10.1177/2057150X211002981

[ref46] Okun M. A. Stock W. A. Haring M. J. Witter R. A. (1984). Health and subjective well-being: a meta-analyis. Int. J. Aging Hum. Dev. 19, 111–132. doi: 10.2190/QGJN-0N81-5957-HAQD, PMID: 6519817

[ref47] Olivia R. Jessica B. (2023). International Women’s day: global opinion remains committed to gender equality, but half now believe it is coming at the expense of men. Available at: https://www.ipsos.com/en/international-womens-day-global-opinion-remains-committed-gender-equality-half-now-believe-it (Accessed April 14, 2024).

[ref48] Qian Y. Li J. (2020). Separating spheres: cohort differences in gender attitudes about work and family in China. China Rev. 20, 19–52.

[ref49] Seol K. O. Kim J. Baek S. (2022). Women’s relative earnings and depressive symptoms in dual-earner households in South Korea: traditional gender role attitudes as moderators. Psych J. 11, 520–529. doi: 10.1002/pchj.554 35413749

[ref50] Shu X. Chen J. Zhu Y. (2023). Changing times and subjective well-being in urban China 2003–2013: An age-period-cohort approach. Chin. J. Sociol. 9, 321–354. doi: 10.1177/2057150X231180022

[ref51] Stevens J. (1992). Applied multivariate statistics for the social sciences. Hillsdale, NJ, US: Lawrence Erlbaum Associates.

[ref52] Sun Y.. (2022). Gender conflict in China in the context of new media, in Proceedings of the 2021 International Conference on Social Development and Media Communication (SDMC 2021), (Atlantis Press), 1385–1389.

[ref53] Sun S. Seo M. (2022). Socioeconomic status, communication activity patterns, and subjective well-being: evidence from a nationally representative sample in China. Anal. Soc. Issues Public Policy 22, 735–757. doi: 10.1111/asap.12319

[ref54] Taniguchi H. Kaufman G. (2014). Gender role attitudes, troubles talk, and marital satisfaction in Japan. J. Soc. Pers. Relatsh. 31, 975–994. doi: 10.1177/0265407513516559

[ref55] United Nations Development Programme (2023). 2023 gender social norms index (GSNI): breaking down gender biases: shifting social norms towards gender equality. New York, NY, US: United Nations.

[ref56] van de Vijver F. J. R. (2007). Cultural and gender differences in gender-role beliefs, sharing household task and child-care responsibilities, and well-being among immigrants and majority members in the Netherlands. Sex Roles 57, 813–824. doi: 10.1007/s11199-007-9316-z

[ref57] van der Horst M. (2014). “Gender role attitudes” in Encyclopedia of quality of life and well-being research. ed. Michalos A. C. (Dordrecht: Springer Netherlands), 2451–2453.

[ref58] Walter J. G. (2018). The adequacy of measures of gender roles attitudes: a review of current measures in omnibus surveys. Qual. Quant. 52, 829–848. doi: 10.1007/s11135-017-0491-x, PMID: 29568133 PMC5847156

[ref59] Wang Y. (2021). A study of factors influencing happiness from the perspective of feminism. China Econ. 33, 224–226.

[ref60] Wang R. A. H. Haworth C. M. A. Ren Q. (2022). Keeping up with the Wangs: individual and contextual influences on mental wellbeing and depressive symptoms in China. BMC Public Health 22:611. doi: 10.1186/s12889-022-12869-8, PMID: 35351074 PMC8962056

[ref61] Wang P. VanderWeele T. J. (2011). Empirical research on factors related to the subjective well-being of Chinese urban residents. Soc. Indic. Res. 101, 447–459. doi: 10.1007/s11205-010-9663-y, PMID: 21731171 PMC3128377

[ref62] Wu Y. Cao Y. (2023). Research on the influence of ecological environment satisfaction and income level on Chinese residents’ happiness: empirical analysis based on CGSS data. Sustain. For. 15:8175. doi: 10.3390/su15108175

[ref63] Wu Y. Wang J. Wang X. (2022). Changes in Chinese Men’s and Women’s gender role attitudes, 1990-2018: a dynamics analysis of the age-period cohort effect. J. China Women Univ. 34, 76–90. doi: 10.13277/j.cnki.jcwu.2022.04.009

[ref64] Xu J. Liu A. (2021). Family life and Chinese adults’ happiness across the life span. Chin. J. Sociol. 7, 514–534. doi: 10.1177/2057150X211045484

[ref65] Yan Y. (2003). Private life under socialism: love, intimacy, and family change in a Chinese Village, 1949–1999. Stanford, CA, US: Stanford University Press.

[ref66] Yang J. (2017). Continuity and change in Chinese gender concepts over the past 20 years. Shandong Soc. Sci. 31, 60–71. doi: 10.14112/j.cnki.37-1053/c.2017.11.009

[ref67] Zhang H. (2015). Wives’ relative income and marital quality in urban China: gender role attitudes as a moderator. J. Comp. Fam. Stud. 46, 203–220. doi: 10.3138/jcfs.46.2.203

[ref68] Zhang W. (2022). Social capital, income and subjective well-being: evidence in rural China. Heliyon 8:e08705. doi: 10.1016/j.heliyon.2021.e08705 35028475 PMC8741508

[ref69] Zhang Y. Liu H. (2022). Individual’s gender ideology and happiness in China. Chin. Sociol. Rev. 54, 252–277. doi: 10.1080/21620555.2021.1871727, PMID: 35814530 PMC9268205

[ref70] Zhang J. Xie J. Zhang X. Yang J. (2022a). Income, social capital, and subjective well-being of residents in Western China. Sustain. For. 14:9141. doi: 10.3390/su14159141

[ref71] Zhang J. Zuo X. Yu C. Lian Q. Zhong X. Tu X.. (2022b). Association of Gender Role Attitudes with adolescent depression. Chin. J. of Sch. Health 43, 181–184. doi: 10.16835/j.cnki.1000-9817.2022.02.005

[ref72] Zhao K. Zhang M. Kraimer M. L. Yang B. (2019). Source attribution matters: mediation and moderation effects in the relationship between work-to-family conflict and job satisfaction. J. Organ. Behav. 40, 492–505. doi: 10.1002/job.2345

[ref73] Zhaopin. (2022). 2022 survey on the current situation of Chinese women in the workplace. Beijing, China: Zhaopin.

[ref74] Zuo J. (2002). Viewing the inequality between husband and wife in Chinese cities from a diversified angle. J. Chin. Women Stud. 11, 12–22.

